# Methodology for automated greenhouse gas data collection and transmission to cloud repository using low-cost drones

**DOI:** 10.1007/s10661-026-15478-9

**Published:** 2026-05-26

**Authors:** Antonio Carlos Daud Filho, Glauco Augusto de Paula Caurin, José Reinaldo Silva, Elinilson Vital, Emilio Carlos Nelli Silva

**Affiliations:** 1 Department of Mechatronics and Mechanical Systems Engineering, Polytechnic School, University of São Paulo, Av. Professor Mello Moraes, 2231, São Paulo, 05508-030 São Paulo Brazil; 2 Department of Aeronautical Engineering, São Carlos School of Engineering, University of São Paulo, Av. João Dagnone, 1100, São Carlos, 13566-590 São Paulo Brazil; 3 Research Centre for Greenhouse Gas Innovation, RCGI, Av. Professor Mello Morais, 2231, São Paulo, 05508-030 São Paulo Brazil

**Keywords:** GHG data, Environmental monitoring, Low-cost drones, Data space, Cloud service

## Abstract

Greenhouse gas (GHG) emissions are crucial for monitoring and mitigating climate change and the degradation of sensitive biomes. Such demand motivates the search for automated processes to collect and manage GHG emissions data, with open access to researchers and institutions working with sustainability. This work proposes an automated data collection process using low-cost drones, with direct data transfer to a cloud-based data space. Low-cost drones were equipped with onboard sensors to measure $\boldsymbol{CO}_{\boldsymbol{2}}$ and methane emissions. The focus was not on data accuracy but on automating data collection and transmission, drone design specifications, and testing, exploring the balance between data accuracy and low-cost sensors. The first practical proof-of-concept experiments demonstrating the system’s capabilities used a drone prototype with simple sensors in an outdoor campus environment, sending data to a cloud-based data space called Digital Amazon (intended to store GHG emissions from the Amazon Forest), via 4G internet communication network. The system’s design addressed aspects such as avoiding interference during data collection and trajectory adjustment, data transfer, and finalizing dataset composition in the cloud. The results provide initial evidence supporting the feasibility of the proposed system in an outdoor environment. However, its application to more complex scenarios, such as forests, other biomes, or urban areas, will be explored in subsequent research based on the reference model presented and will require further validation under diverse environmental and operational conditions. Enhancements to accommodate future communication based on Low Earth Orbit (LEO) and Very Low Earth Orbit (VLEO) satellite systems would help reduce transmission latency, but this issue was not assessed in the present study.

## Introduction

Greenhouse gas (GHG) monitoring has become a widespread demand, driven by the need to mitigate climate change (Schaeffer et al., 2025) and achieve sustainability in various activities to preserve the ecosystem. One possible cause of these adverse climate events is the emission of greenhouse gases into the atmosphere emerging from industrial processes, the burning of fossil fuels, and uncontrolled deforestation. In Brazil, forest fires in the Amazon rainforest also contribute significantly to greenhouse gas emissions. Official forest degradation alerts for Brazilian Amazon in 2024—including scars from forest fires, logging, and other forms of forest degradation not related to drought—reached 25,023 $km^2$, a 44% increase compared to 2023 (17,473 $km^2$) and 163% compared to 2022 (9549 $km^2$) (Mataveli et al., 2025).

In the Amazon forest, GHG emission data can be captured using remote sensing, land-based towers such as the Amazon Tall Tower Observatory (ATTO tower) (Andreae et al., 2015), or those belonging to the Large-Scale Biosphere-Atmosphere Experiment in Amazonia (LBA) (de Gonçalves et al., 2013). Remote sensing is very efficient but struggles to process data at different altitudes, mainly due to cloud cover. Using a land-based tower can collect this data over a wide area, but the tower’s maximum acquisition range limits data collection. A tall tower provides the unique ability to obtain continuous measurements at a series of heights throughout the lower part of the planetary boundary layer. Investigations of phenomena such as the formation and dissolution of nocturnal stable boundary layers, the production and behavior of intermittent turbulent structures, and boundary layer roll (Andreae et al., 2015) can benefit from this approach. The measurement of wind speed and direction using sensors such as a 3-D sonic anemometer, mean wind speed, and wind direction sensor installed on the towers allows the application of the eddy covariance method to calculate gas emission and consumption rates, allowing measurements of momentum, sensible heat, and latent heat fluxes integrated over areas of various sizes (Burba, 2013; Burba et al., 2013). However, fixed structures have the disadvantage of providing measurements of interest from a specific region.

Sensors onboard a human-crewed aircraft offer advantages over those embarked on unmanned aerial vehicles (UAVs) for collecting atmospheric data: they possess higher payload capacity, faster flight speeds for broad-area coverage, and longer flight time. In the literature, Sun et al. (2023) developed a monitoring and sampling platform using helicopters to monitor air quality in real time on a routine basis. The ACRIDICON–CHUVA campaign (Wendisch et al., 2016) used an airplane whose primary objective was to quantify the influence of aerosol particles and trace gases (natural and anthropogenic) on cloud evolution and precipitation formation, as well as the cloud thermodynamic, dynamic, and radiative effects in the Amazon region. For this purpose, the German High Altitude and Long Range Research Aircraft (HALO), an ultra-long-range Gulfstream G550 business jet, with a ceiling altitude of up to 15 km and endurance of up to 8 h, hosted the sensors. Thus, despite being a very effective means of measuring greenhouse gas parameters, it is also expensive and demanding sophisticated operation.

Satellite observations can provide measurements of the column-averaged dry-air mole fraction of $CO_{2}$, widely used in environmental monitoring. Although it requires substantial initial costs for launch and maintenance, the average cost per emission source or sampled pixel is lower than that of conventional bottom-up methods (Pan et al., 2021). The disadvantage of this method is its spatial resolution, which is on the order of kilometers, and its temporal cycle, which is on the order of days, compromising a more detailed regional analysis of GHG emissions dynamics.

GHG emissions compelled the government and industry to seek cost-effective, systematic methods for monitoring (Burgués & Marco, 2020). For instance, Bonne et al. (2024) have developed a tool to quantify $CO_{2}$ and $CH_{4}$ emissions at an industrial site, based on a mass balance approach that relies on a newly developed lightweight open-path laser absorption spectrometer, operable on UAVs. High spatial-resolution atmospheric concentrations enable the derivation of a plume cross-section downwind of a source within the limited UAV flight period, thereby quantifying emissions. Another essential issue is detecting and locating gas leaks, such as methane (Allen et al., 2019). In Golston et al. (2018), the authors apply a method of rotating the methane reading data from a small UAV around the estimated leak location according to the wind, with the leak magnitude calculated from the average crosswind integrated flux in the region near the source location.

Gas concentration data acquisition is also used to monitor emissions or absorptions in a wide range of ecosystems and biomes, including forested, mountainous, and rural environments. Simultaneously measuring wind speed and GHG concentration with sensors on board or with a wind speed sensor positioned on the ground, such as the Lidar sensor (Bonne et al., 2024), allows the calculation of the local gas flow using the mass balance method which derives the net flux, by integrating the concentration (above background level) across a vertical sampling plane downwind of the emitting source, and multiplying the result by the wind speed perpendicular to that plane (Allen et al., 2019; Burgués & Marco, 2020). Furthermore, the net ecosystem exchange (NEE) of a region can be calculated over a given period using GHG measurements at varying heights at different times (Kunz et al., 2020) by integrating the continuity equation along the height profile of the measurements. Atmospheric air samples can also be collected using bags carried on the UAV, filled by pumps. The pumps are activated at different altitudes for later laboratory analysis using high-precision equipment that cannot be carried on the UAV due to its size and weight (Zhou et al., 2025), such as a gas column chromatograph coupled to a mass spectrometer. Furthermore, the combination of unmanned vehicles, such as watercraft, multicopters, airships, and aircraft, has the potential to monitor across a wide range of altitudes (Caurin & Daud Filho, 2023).

Automating GHG Data Collection requires a clear mission definition and a flight path that balances UAV flight-time limitations with the required detection at different heights. A better approach is to define a zigzag/spiral flight path that scans the broadest possible area of emissions, since GHG emissions from the biomass considered in this work do not necessarily follow a Gaussian plume (Draxler, 1981). Fitting the dispersion mode for these areas results in data collection over a wider area rather than an influence circle around the emission source (Afshar-Mohajer & Wu, 2023).

The limited payload capacity of small UAVs requires lightweight and low-power instrumentation (Motlagh et al., 2023). Popular options for UAV applications include GHG sensors such as amperometric gas sensors (AGS), metal-oxide-semiconductor (MOS) sensors, non-dispersive infrared (NDIR) sensors, and photoionization detectors (PIDs) (Burgués & Marco, 2020). The primary greenhouse gases of interest are carbon dioxide ($CO_2$), methane ($CH_4$), nitrogen oxides ($NO_x$), and sulfur oxides ($SO_x$). In addition to gas concentration sensors, there is interest in sensors for measuring temperature, relative humidity, atmospheric pressure, particulate matter (Guerrón et al., 2023), and wind speed, both on the drone (Zhang et al., 2025) and at a ground-based weather station. Sensor positioning on the UAV’s body is also an important issue: placing them beneath the multicopter’s structure results in turbulent air from the propellers being pulled from regions above the aircraft and pushed downwards. In practice, $CO_{2}$ concentration is due to a height slightly above a multicopter. Flying close to the ground generates air turbulence at the earth’s surface, and vorticity can raise layers of $CO_{2}$, interfering with gas concentration measurements. Therefore, below 9.0 m, it is best to measure manually or with masts (Kunz et al., 2020). The multicopter’s ascent and descent can also influence the measurement of GHG concentration, since the airflow around the multicopter is modified. The movement that suffers the least interference is horizontal flight, since it allows the sensor to be positioned outside the propeller vortex trail.

The transmission of data collected by UAVs to the cloud repository is also a challenge, especially in remote areas such as the Amazon rainforest. A wireless connection is required to transmit data, varying from satellite communication to a simple internet connection. In our proof of concept, the internet was the option, based on a 4G/5G ground public network available in most urban environments and populated regions. The focus was on automating GHG data collection and exploring alternatives for composing packages and sending them directly to a cloud repository. Completing the entire process also provides feedback on latency, but the primary focus was on data management and reducing package loss.

In practice, a satellite connection may be the only option for data transmission in remote environments such as forests or petroleum platforms. However, in urban areas, large plantations, or even forest regions near cities, IP connections are feasible with signal amplifiers.

The drone’s continuous connection to the internet, and consequently the near-real-time transmission of measured data, allows simultaneous analyses and evaluations for the monitoring of the environment, as well as the application of path planning algorithms for autonomous aerial exploration, and the use of algorithms to identify and locate gas emission sources (Francis et al., 2022; Hutchinson et al., 2019), whose division of tasks can be shared between the edge computing embedded in the drone, and cloud computing (Hayat et al., 2021; Liu et al., 2019).

The development of this type of automated GHG data collection and transmission system could make it possible not only to monitor the environmental flow of gases in forests, but also to assist in combating forest and plantation fires by detecting and locating emission sources, via algorithms such as particle filters (Hutchinson et al., 2019) and machine learning tools (Badawi et al., 2022; Zhao et al., 2022).

Forest watching is a primary objective of GHG monitoring, particularly in the Amazon rainforest, which has enormous potential for global carbon sequestration. The current proposal aims to amplify the potential and agility for monitoring and capturing GHG emissions across other biomes, large farms, offshore petroleum facilities, and urban regions by introducing UAVs directly connected to a cloud data space. The reference design model focused on defining the essential UAV requirements to capture data without interference, complete the mission on a reasonable schedule, and securely transfer data to the cloud.

A practical approach addresses the entire mission, overcoming technological barriers to achieve an automated, agile data-capture process. The first barrier concerns the design of a VTOL (vertical takeoff and landing) UAV to transport reliable GHG sensors and their batteries. The second barrier concerns adding communication capacity to transfer data with low latency and reduce packet loss. The third barrier concerns providing a secure and reliable transfer to the cloud data repository.

The ultimate goal is to consolidate the proposed method by developing a prototype that can fly 100 km from the starting point and return, carrying all necessary sensors, thereby completing its mission. Further developments can involve reducing the payload through advances in accurate sensors or by investing in hydrogen fuel cells, making the model even more sustainable. Regarding the Amazon Forest, the collection process relies on a vast river basin (one of the largest in the world), which enables the placement of the drone in strategic locations accessible by boat, thereby eliminating the need for drone relocation. The application to other biomes would rely on short missions; therefore, we focus the current work on a safe collection and data transfer.

The flexibility of UAV missions results from integrating high coverage with reduced collection time, providing good data availability by managing latency. Cloud systems can offer flexibility in direct missions to specific locations on demand, based on the data collected. Therefore, the current work focuses on the reliability of the collecting process and on the integrity and latency of data transfer to the cloud. Experiments used low-cost sensors to find a good balance between cost and accuracy, which varies across different collection environments. However, this topic requires further consideration to achieve higher accuracy with more advanced sensors that reduce the payload. Recent advances in the academy and industry are overcoming this barrier.

In less than 5 years, the industry has reduced the weight of the most accurate sensors for GHG emissions by more than 50%, and further reductions are expected in the coming years. Therefore, the challenge now is to integrate secure collection with reliable and agile data transfer. The first goal is to adjust the collected data with the package size to optimize the transfer.

The continuous acquisition of greenhouse gas data across regions requires tools for storing, processing, and managing the data. To this end, a cloud data space called Digital Amazon (https://www.rcggi.org) and appropriate APIs (Application Programming Interface) were developed to operate the transfer, reception, and encapsulation of large amounts of data in a suitable data space (Silva et al., 2023).

**Fig. 1:**
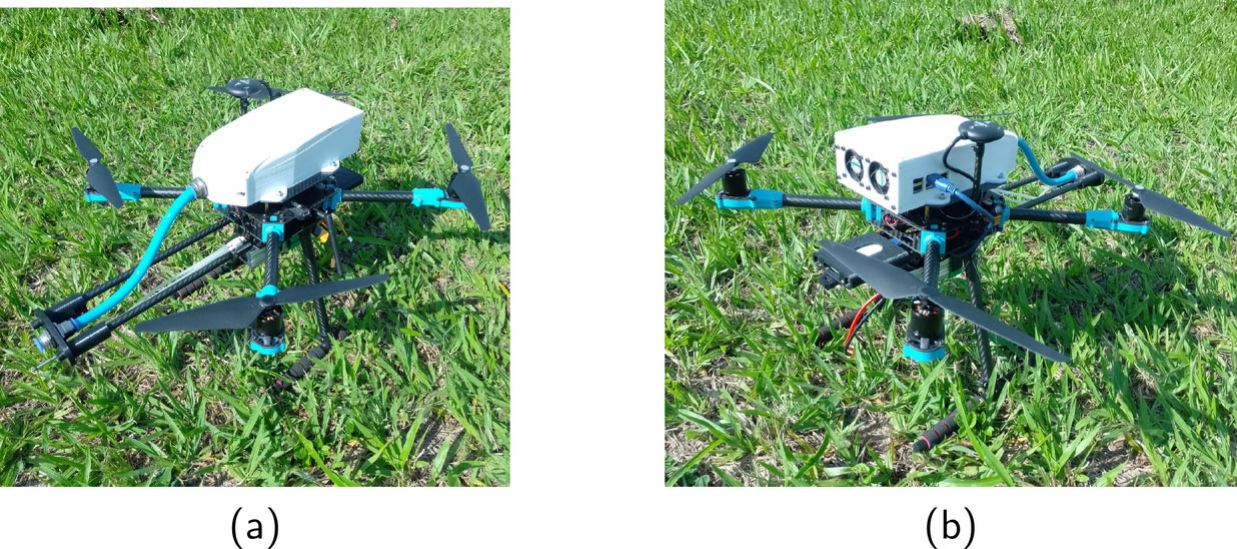
Drone developed to measure greenhouse gases. **a** Front view, **b** rear view

The main contributions of this work are as follows:

- Development of a low-cost drone-based system equipped with sensors for greenhouse gas measurement and environmental monitoring, but also the establishment of a reference framework for integrating such systems in non-stationary, flight-based missions.
- Definition of a methodology for automating GHG data collection, which incorporates in-flight GHG data acquisition, processing, and transmission to a cloud-based data repository via 4G wireless communication during flight, adaptable to satellite-based communication when there is no wireless infrastructure.

**Table 1:** Low-cost drone specifications

Drone frame	Holybro X500 V2
Onboard computer	Raspberry Pi 4B
Flight controller	Pixhawk 6C
Flight controller firmware	PX4
Battery	Lipo 4S 6500 mAh
Wifi-modem	4G LTE
Total weight	2.4 kg
Flight time	8.0 min
$CO_{2}$ sensor	Senseair K30 FR
$CH_{4}$ sensor	Winsen MH-441D NDIR
Relative humidity and temperature sensor	DHT22

**Fig. 2:**
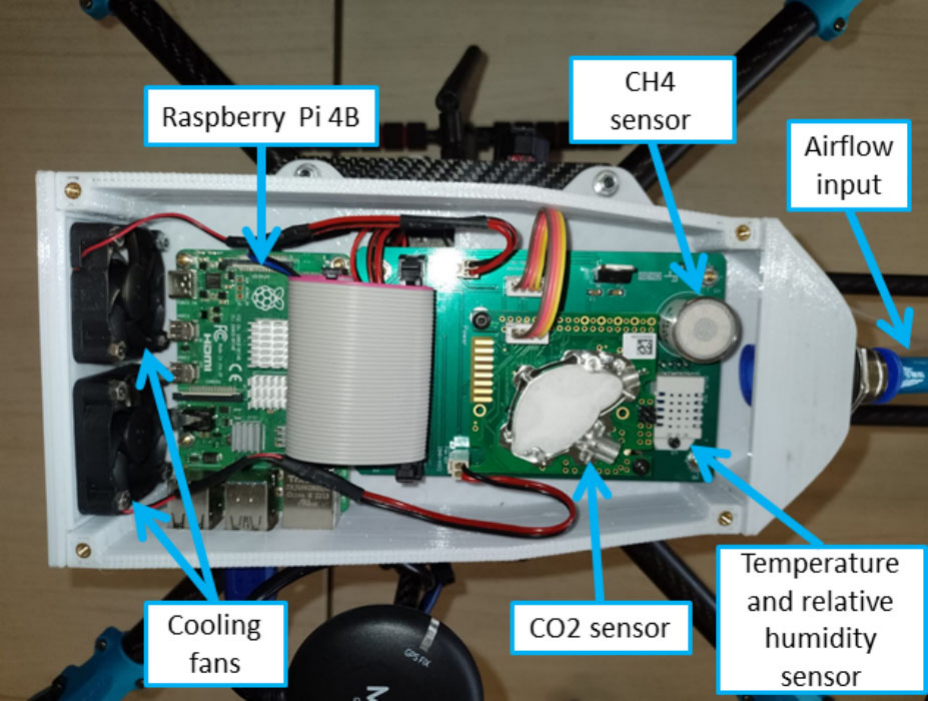
View of the drone sensors chamber

**Table 2:** GHG data low-cost sensor specifications

Sensor	Measurement	Operating principle	Measurement range	Accuracy
SenseAir K30 FR	$CO_2$ (ppm)	NDIR	0–5000 $ppm_{vol}$	± 30 ± 3% of reading
Winsen MH-441D	$CH_4$ (ppm)	NDIR	0–50,000 $ppm_{vol}$	± (50 ppm + 5% reading value)
DHT22	Temp. ($^{\underline{\text {o}}}$C) and Rel. Hum. (%)	Polymer humidity capacitor	−40 to 80$^{\underline{\text {o}}}$C ; 0–100%	Temp.: ± 0.5$^{\underline{\text {o}}}$C; Rel. Hum.: ± 2%
MS5611	Atmospheric Pressure	Piezo-resistive pressure sensor	10–1200 mbar	± 1.5 mbar

The article’s further organization is as follows: the “Drone architecture and prototype” section presents the drone architecture used in the experiments and the features that enable reliable collection. The “Data processing” section describes the data processing and sensor calibration procedure. The “The automated collection process” section will describe the automatic collection process, combining data collection and data transfer. The “Current test results” section presents the current experimental results, followed by a discussion (Section “Discussion”). Finally, the “Conclusion” section presents the concluding remarks.

## Drone architecture and prototype

We based automatic drone collection on a Holybro X500 V2 quadcopter platform, which allows adaptations and modifications, such as adding sensors and microprocessor boards to carry out missions of interest. The model has a Pixhawk 6C flight controller board with the PX4 autopilot firmware.^1^ The model includes a sensor chamber built to accommodate the sensors. A printed circuit board installs the sensors and enables communication with the drone’s onboard computer, a Raspberry Pi 4B with Raspberry Pi OS. Additionally, the drone has a 4G LTE Wi-Fi modem. The final result is shown in Fig. 1, and Table 1 lists the drone specifications.

Figure 2 shows the interior of the sensor chamber, with the top cover removed. The sensor chamber has an air inlet at the front, which is in the direction of flight. A hose can be attached to this air inlet. The device design avoids turbulent airflow from the propellers, which pushes air mass from layers above the drone. In other words, the propellers push the air downwards to produce upward traction force, producing a constant airflow from the air layers above the drone in a downward direction. Therefore, a hose collects the air sample at the same altitude during the flight, while the sensor chamber has an air outlet with fans that produce a constant airflow inside the chamber. During horizontal flight, the rotor-induced airflow should have a minor effect on measurements because the sampling system is moving away from the displaced air (Kunz et al., 2020).

The onboard sensors include a Senseair K30 FR, a $CO_{2}$ sensor module optimized for fast response time using non-dispersive infrared (NDIR), and a particle filter that protects the sample cell and enables fast diffusion without an external pump. A Winsen MH-441D NDIR $CH_{4}$ sensor module, a DHT22 digital relative humidity and temperature sensor whose sensing element, is a polymer humidity capacitor. The flight controller’s barometer, MS5611, measures the atmospheric pressure. Table 2 lists the GHG data low-cost sensor specifications.

## Data processing

Calibration is a challenge with low-cost sensors. In our experiment, we used a preceding zero calibration procedure, which involves positioning the sensors in a deep, soft plastic bag and flushing nitrogen reference gas into the bag. Nitrogen gas with a purity of 99.95% was used. The readings are monitored, and once they stabilize at values near zero (0–5 ppm), with an oscillation of only ± 1 ppm, the command to calculate the zero measurement point is triggered. If the sensor is giving readings that are far from the expected value, for example, readings far from 400 ppm in an outdoor environment, far from disturbances and emission sources, the SenseAir K30FR sensor allows for an initial calibration to a $CO_2$ concentration reference value of 400 ppm. This can be done using a reference cylinder with $CO_2$ at 400 ppm, or by positioning the sensor outdoors to measure approximately 400 ppm. Following this, perform a zero calibration using nitrogen gas.

According to the manufacturer’s manual for the SenseAir K30 FR sensor, the sensors must be recalibrated before important measurements are taken, and that is exactly what was done for the experiments described in this article. The sensor automatically compensates for temperature effects but not for relative humidity. Atmospheric $CO_2$ concentrations measured in moist air must be corrected for the influence of water vapor, which primarily affects the reported values through dilution. In a gas mixture, the mole fraction of $CO_2$ is defined relative to the total number of moles, including water vapor. Therefore, the presence of $H_2O$ reduces the apparent $CO_2$ mole fraction when expressed on a wet basis. To obtain dry-air mole fractions—standard in atmospheric studies—the measured value must be adjusted by removing the contribution of water vapor. Assuming ideal gas behavior and applying Dalton’s law, the dry mole fraction of $CO_2$ can be expressed as

1 $$\begin{aligned} x_{CO_2, dry} = \frac{x_{CO_2, wet}}{1 - x_{H_2O}} \end{aligned}$$

where $x_{H_2O}$ is the mole fraction of water vapor (Reum et al., 2019). When relative humidity and temperature are available, $x_{H_2O}$ can be estimated from the ratio of water vapor partial pressure to total pressure, with the partial pressure derived from relative humidity and saturation vapor pressure (Buck, 1981; Murray, 1966).

2 $$\begin{aligned} x_{H_2O}=\frac{RH}{100}\frac{e_s(T)}{P} \end{aligned}$$

Being that *RH* is the relative humidity, $e_s(T)$ is the saturation vapor pressure in *hPa*, *P* is the total atmospheric pressure in *hPa*, and *T* is the temperature in degrees Celsius. According to Buck (1981), the saturation vapor pressure can be expressed as

3 $$\begin{aligned} e_s(T)=6.1121 \exp \left( \frac{17.502T}{240.97+T} \right) \end{aligned}$$

A conversion of the mole fraction to ppm is achieved by multiplying by $10^6$, resulting in the water vapor correction equation

4 $$\begin{aligned} CO'_{2,dry}(ppm)=\frac{CO_{2,wet}(ppm)}{1-\frac{RH}{100}\frac{e_s(T)}{P}} \end{aligned}$$

This correction is essential for ensuring comparability of $CO_2$ measurements across varying environmental conditions and is widely adopted in atmospheric monitoring and greenhouse gas quantification studies (Martin et al., 2017).

**Fig. 3:**
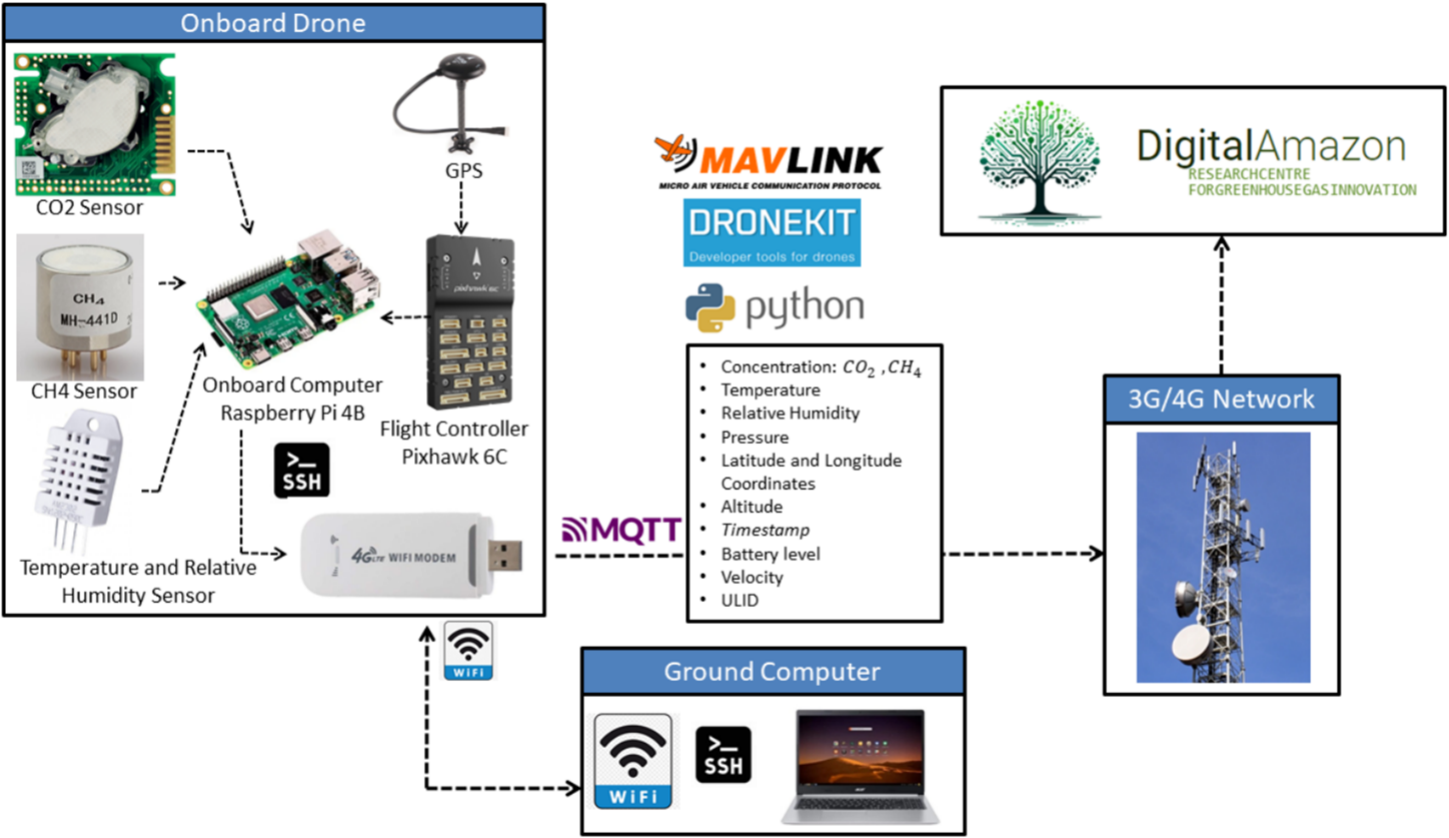
System architecture of the communication, data acquisition, and transmission to the Digital Amazon cloud data repository

Additionally, $CO_{2}$ concentration measurements by the SenseAir K30 FR sensor are corrected by the calibration curve described in Martin et al. (2017), which corrects span and zero offset relative to a research-grade analyzer, the LGR-24A-FGGA fast greenhouse gas analyzer from Los Gatos Research (LGR), via the following equation. This provides indirect validation against a reference instrument, although no co-located validation was performed in the present experiments.

5 $$\begin{aligned} CO_{2,dry}(ppm)=\frac{CO'_{2,dry}(ppm) - 31.5841}{0.9395} \end{aligned}$$

The Winsen MH-441D methane gas sensor has a resolution of 100 ppm. Therefore, it cannot measure background methane concentrations. According to Vallero (2025), the composition of unpolluted air in the atmosphere contains methane gas at approximately 1.0 to 1.2 $ppm_{vol}$ (dry basis). This sensor can measure concentrations above 100 ppm, i.e., it is intended only to detect high levels of this gas from emission sources and leaks.

## The automated collection process

The automation process integrates drone GHG collection with automatic cloud data transfer, eliminating manual data transfer. Another great advantage comes from a significant reduction in package loss and in the data management provided by Digital Amazon, which comprises datasets indexed by metadata. The process flow is illustrated in Fig. 3.

A Python code was written to fulfill the tasks of algorithm 1. Once there is an internet connection available, either via 4G network, or satellite bases, it establishes a connection with the onboard computer (Raspberry Pi 4B) and the flight controller (Pixhawk 6C) via Dronekit-Python,^2^ as well as with onboard sensors ($CO_2$, $CH_4$, temperature, relative humidity). It also establishes a connection to the AWS cloud. A flight path must be defined, and before starting the mission, a unique digital identifier ULID^3^ must be generated, as well as a CSV file to store backup data in the onboard computer’s memory. As the mission starts, the UAV takes off, and the main loop executes, with the data obtained synchronized by reading the current time, stamping it with the ULID, and saving it sequentially in the CSV file sent to the cloud via MQTT. Data transmission rate is 1.0 Hz.

**Algorithm 1:**
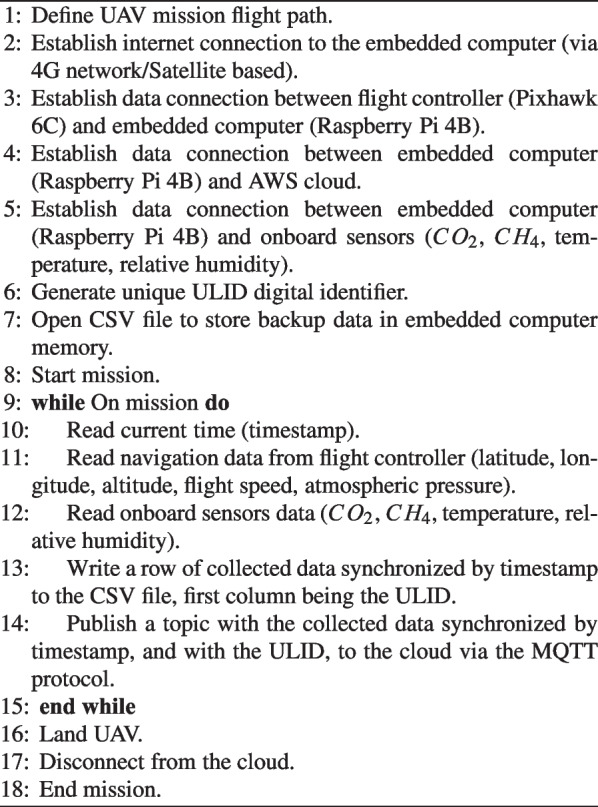
Process for GHG data acquisition and transmission to the cloud.

Each onboard sensor takes measurements at a specific frequency, but the last measurement remains available for the onboard computer to read until a new one is taken. In this sense, the Python code running on the drone’s onboard computer begins by generating a ULID (Universal Unique Lexicographically Sortable Identifier) for that single experiment, then reads and saves the current time (month/date/year hour:minute:second). Following that, it takes the available readings from each sensor and saves the dataset, synchronized with the start time of the reading loop. This package of timestamp, sensor data, and ULID is saved in the onboard computer’s memory and transmitted to the cloud repository. The delay due to air traveling through the tube could not be measured. The data packets sent to the cloud repository also include the read timestamps.

**Fig. 4:**
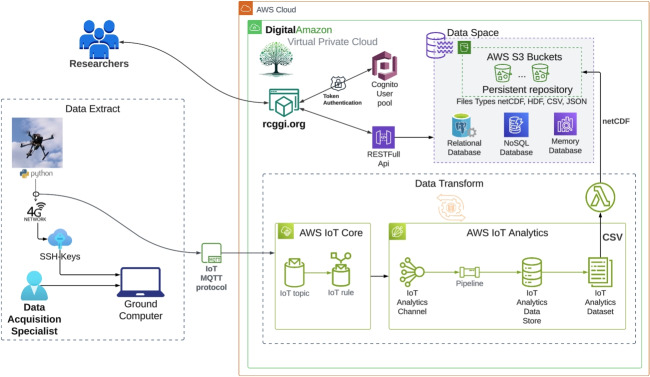
System architecture diagram for data transmission, storage, and analysis to the Digital Amazon in the AWS cloud using a 4G network

The Wi-Fi connection between the drone’s onboard computer and the ground computer is only necessary to initiate operation. The drone and the onboard computer are connected on the ground. The operator develops the mission program. Then, the code for reading, pre-processing, and transmitting data begins. The automated mission then starts, and after that, the Wi-Fi connection between the drone’s onboard computer and the ground computer is no longer needed. Thus, data transmission to the cloud repository is automatically handled via the drone’s 4G modem. If the 4G network connection fails, the data continues to be stored on the UAV’s computer and can be retransmitted later to the cloud repository.

**Fig. 5:**
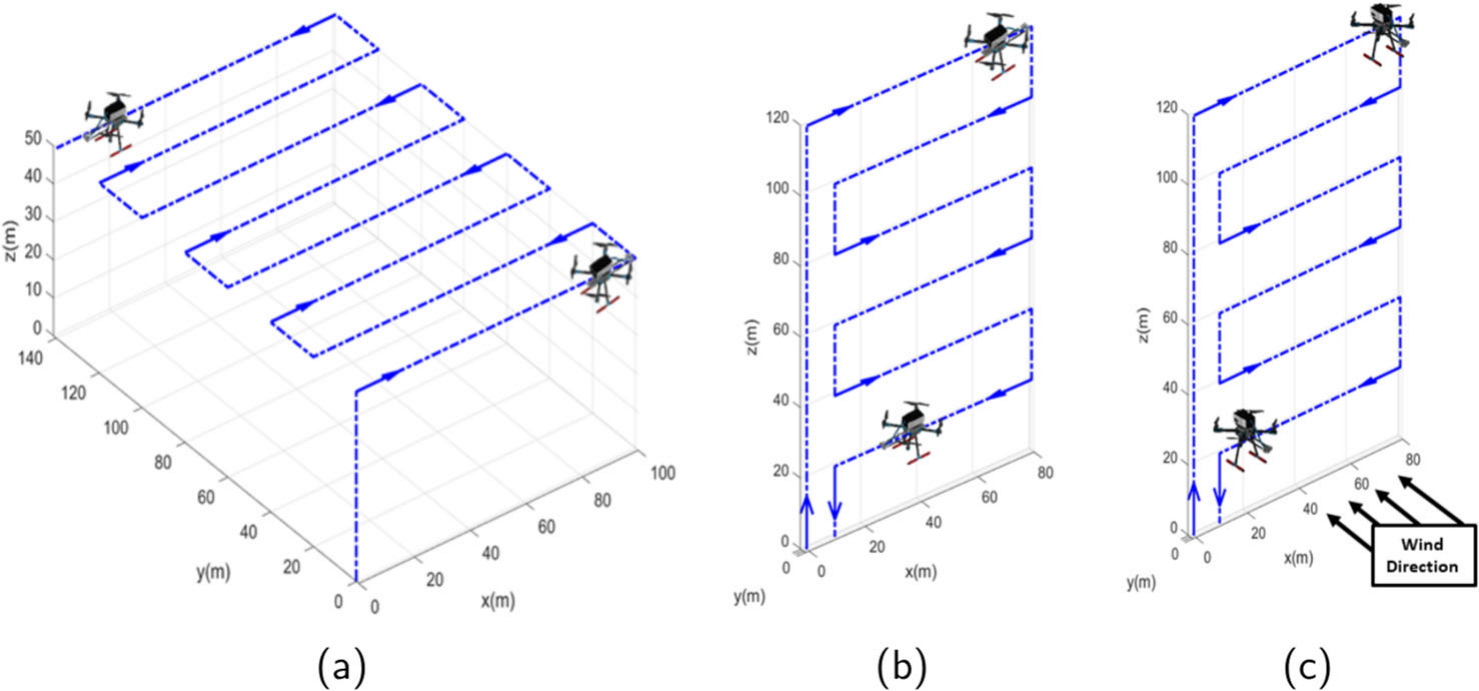
Drone flight path to perform: **a** GHG data mapping. **b** GHG data profile measurement along altitude. **c** Gas flux quantification across the vertical sampling plane

Figure 4 shows the AWS cloud computing network data transmission, storage, and analysis architecture. However, this connection can evolve into an amplified signal system, allowing monitoring over several kilometers in an urban area or a plantation, without changing the basic process. In the future, additional connection options can be added, such as 6G or a direct satellite connection to the VLEOS system. The impact of these communication possibilities will primarily be in latency.

Digital Amazon automatically synthesizes metadata, contextualizing new datasets within the existing data space. The local analytic service processes the incoming data stream, converts it to CSV format, and can transfer the dataset to NetCDF, a widely used format in sustainability research.

Available datasets support the analysis of different user segments and can eventually be combined to create new versions. Another service controls the versions, preserving data sovereignty and intellectual properties. Therefore, the system preserves the original versions of data, and the origin of any derived data can always be traced. We are now enhancing the cloud service to support GRIB, GRIB4 and other formats.

Moreover, each flight test to obtain greenhouse gas data can be retrieved from the repository using its unique digital identifier, generated by the ULID protocol.

The drone flight path must align with the mission-measuring objectives, as shown in Fig. 5: GHG data mapping, GHG data profile measurement along altitude, and gas flux quantification across the vertical sampling plane.

GHG data mapping builds a spatial representation of a given area from spatially distributed sensor measurements (Burgués & Marco, 2020), which helps monitor air quality and locate gas sources. For such a mission, the drone path, shown in Fig. 5a, takes off, reaches a desired altitude level, and follows a sequence of straight paths with equidistant spacing while taking measurements. In the end, it returns to the takeoff point and lands.

GHG data mapping composes a spatial representation for a particular area based on spatially distributed measurements (Burgués & Marco, 2020). The mission follows a simple planning path, shown in Fig. 5a, and described above. However, this planning can be provided dynamically using AI Planning in a subsequent version of this work, thereby accommodating greater flexibility in adapting the mission to different situations.

**Fig. 6:**
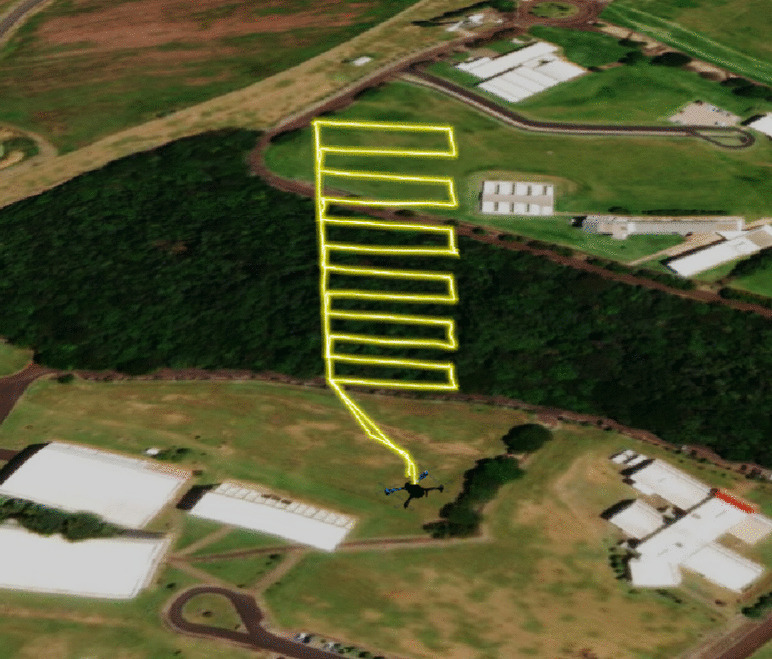
Drone flight path to take measurements profile along altitude

A specific path can be used to compute the environment’s net ecosystem exchange (NEE) by taking measurements along it at different altitudes over time. Thus, the drone must take off and reach a maximum altitude, travel a straight stretch while taking measurements, and descend to a lower level, continuing measurements at subsequent levels, as shown in the sketch in Fig. 5b.

## Current test results

The success of this automation process depends critically on the drone mission and the data transfer to the cloud. After designing the process, the drone, and the data system, we implemented a proof of concept to test GHG collection and data transfer to the Digital Amazon repository. The practical process consists of a drone mission to collect GHG data profiles at different altitudes and build a GHG data mapping over a survey area. The test was implemented at the University of São Paulo (longitude, −47.932853$^{\circ }$; latitude, −22.004291$^{\circ }$).

**Fig. 7:**
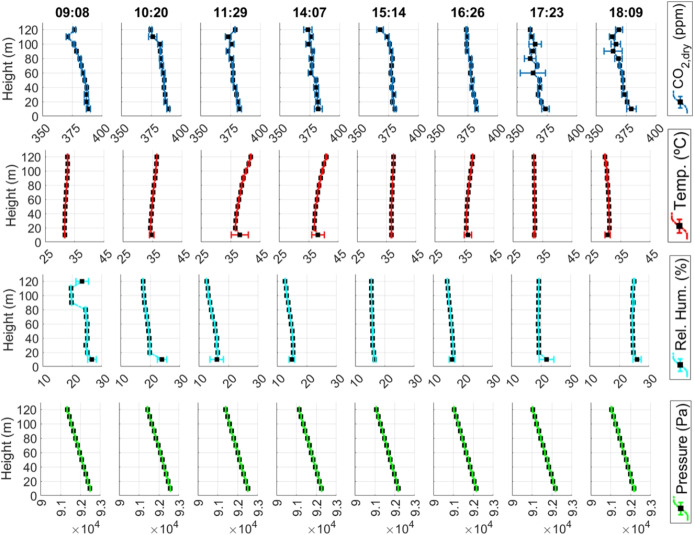
Results of average $CO_{2,dry}$ concentration, temperature, relative humidity, and atmospheric pressure profile along altitude, throughout the day, at the University of São Paulo campus 2 in the city of São Carlos, state of São Paulo, Brazil

### Data profile capture along altitude

Figure 6 shows a drone flight path to test the capacity of measuring GHG data along altitude. Path visualization relied on flight logs on Flight Review.^4^

The planned trajectory has a maximum flight height of 120 m. The drone travels a linear section of 50 m at a speed of 3 m/s with the drone front facing forward, descends 10 m to the lower level of 110 m, travels a linear section of 50 m again at a speed of 3 m/s, and continues this movement down to a height of 10 m and then lands.

We repeated this planned flight several times during the day to detect the effects of different periods on the measured parameters. The drone measures at a continuous height, producing an average value and standard deviation calculated by the edge system. Measurements taken with the drone positioned vertically at different heights are not included in the calculation of average values for each height. Results for a day flight, including the average concentration of $CO_{2,dry}$, temperature, relative humidity, and atmospheric pressure at different flight levels, appear in Fig. 7. For this data profiling survey along the altitude, 8 flights were conducted, and the takeoff times for the same day are shown at the top of the figure. Since there were no sources of methane gas emissions near the experiment site, methane concentration does not appear in this collection.

### Data mapping over the survey area

**Fig. 8:**
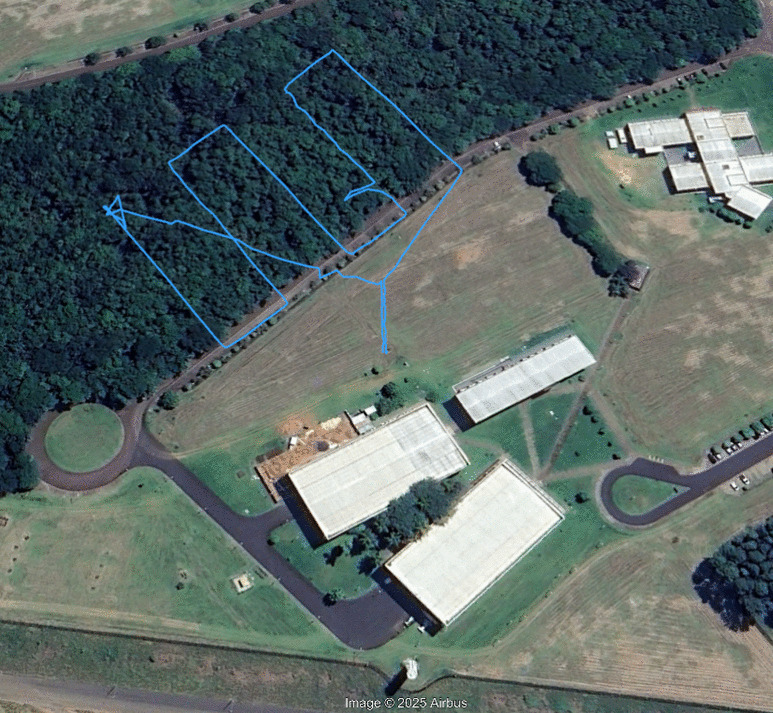
Drone flight path to take GHG data measurements at a height of 50 m

A proof-of-concept mission also tested the capacity to measure GHG emissions over an area at a fixed height (GHG data mapping experiment). The UAV flight path is in Fig. 8.

In this experiment, the UAV trajectory maintained a maximum flight height of 50 m throughout the survey area. Then, the UAV traveled linear segments of 100 m at 3 m/s, with the front segment facing forward and the intermediate segments spaced 35 m apart. It continued this movement until it covered an area of roughly 10500 $m^2$. Notice that the drone deviated from the established trajectory at some moments and returned due to strong wind gusts during data acquisition.

**Fig. 9:**
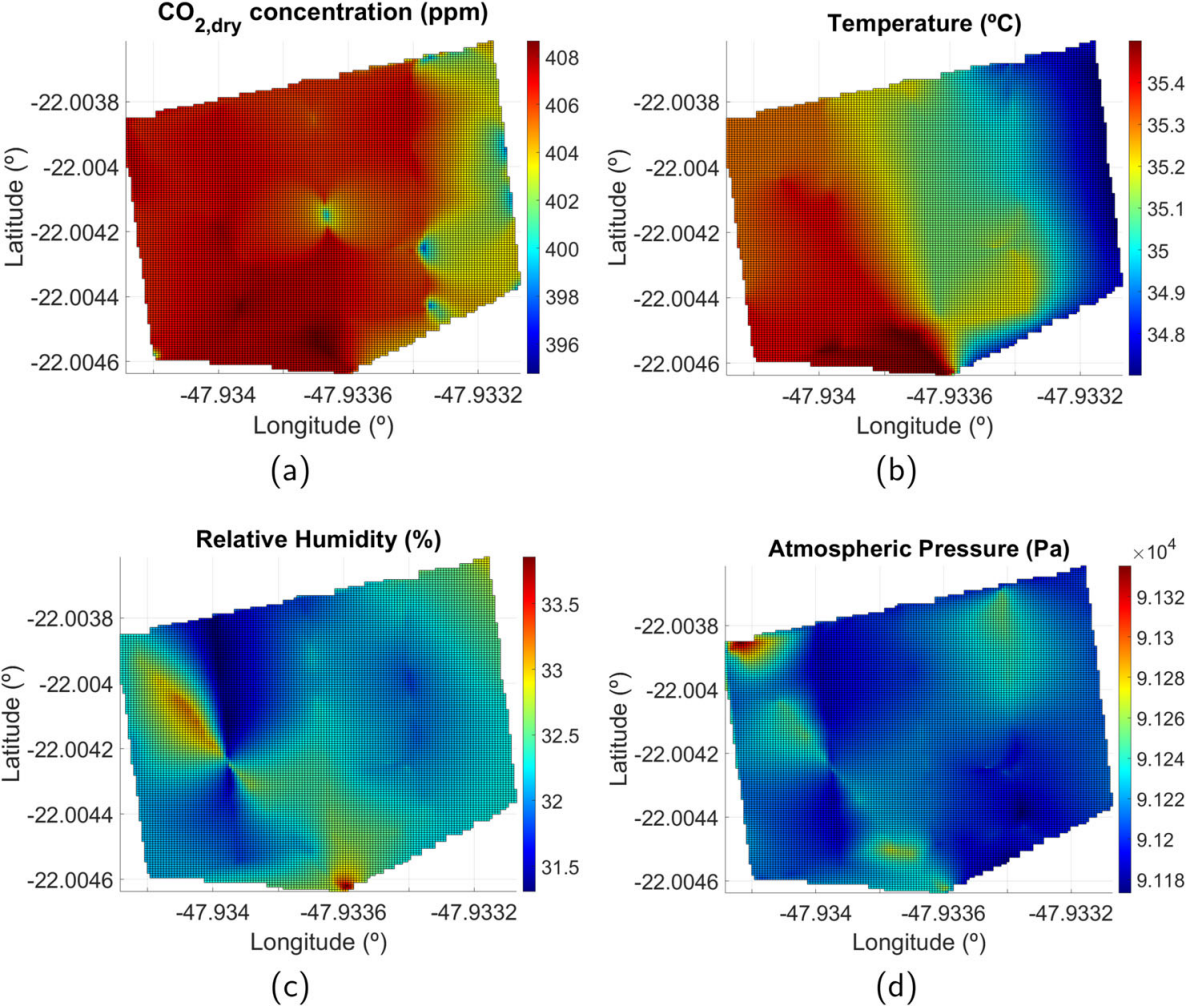
Result of two-dimensional linear interpolations of UAV measurements along the flight path at a height of 50 m. **a** $CO_{2,dry}$ concentration mapping. **b** Temperature mapping. **c** Relative humidity mapping. **d** Atmospheric pressure mapping

Although the data collection follows a linear trajectory, GHG emissions in the survey area can be estimated using two-dimensional linear interpolation. We used MATLAB^5^ to create a grid of 150 $\times$ 150 points along the longitude and latitude axes, equally spaced between the maximum and minimum coordinate points in longitude and latitude. First, the *meshgrid* function was used to create a grid over the measured limits of the latitude and longitude data, and then the *griddata* function was applied to interpolate the scattered points data within the grid. Next, a 150 $\times$ 150-point grid was created using the *meshgrid* function over the data’s latitude and longitude limits. Finally, the *interp2* function was used to interpolate the data from the first grid onto the specific 150 x 150-point grid. The result of the two-dimensional linear interpolation of $CO_{2,dry}$ concentration, temperature, relative humidity, and atmospheric pressure is shown as chart surfaces in Fig. 9, for the same single flight for data acquisition. The results show that $CO_{2,dry}$ concentration varied little across the survey area, ranging from 394.8 to 408.7 ppm. Similar conclusions can be drawn for the other parameters, with slight spatial variation. The reason is that the area studied is relatively small—although large enough to demonstrate the data analysis capabilities—and that there was no nearby source of gas emissions.

**Table 3:** Flight tests data packets transfer performance

Test	Starting time	Ending time	Last packet timestamp in the cloud	Total experiment time (sec)	Total packets	Packets saved in the cloud	Missing packet rate	Avg. loop time (sec/package)
1	09:08:37	09:16:24	09:16:24	467	298	298	0.0%	1.567
2	10:20:40	10:28:42	10:22:29	482	307	71	76.9%	1.570
3	11:29:03	11:36:43	11:36:43	460	293	293	0.0%	1.570
4	14:07:20	14:15:05	14:15:05	465	296	296	0.0%	1.571
5	15:14:06	15:21:23	15:21:23	437	279	279	0.0%	1.566
6	16:26:47	16:34:15	16:34:15	448	284	284	0.0%	1.577
7	17:23:08	17:30:30	17:30:30	442	274	274	0.0%	1.613
8	18:09:01	18:16:47	18:16:47	466	293	293	0.0%	1.590
9	15:48:59	15:56:09	15:56:09	430	274	274	0.0%	1.569

### Data transfer performance

The performance of transmitting sensor data collected to the cloud repository is presented in Table 3. In this list, tests 1 through 8 are for surveying vertical profiles, and test 9 collected data over the survey area. Each experiment took from 7 to 8 min. Each new data packet is sent approximately every 1.57 s. All experiments, except test 2, successfully transmitted all data to the cloud, whereas test 2 showed a 76.9% missing-packet rate when comparing data stored in the cloud with that stored in the computer’s onboard memory. This occurred due to a loss of internet connection at the modem embedded in the UAV during the experiment. Protocols for detecting connection failures and transmitting and resending missing data to the cloud still need to be developed.

**Fig. 10:**
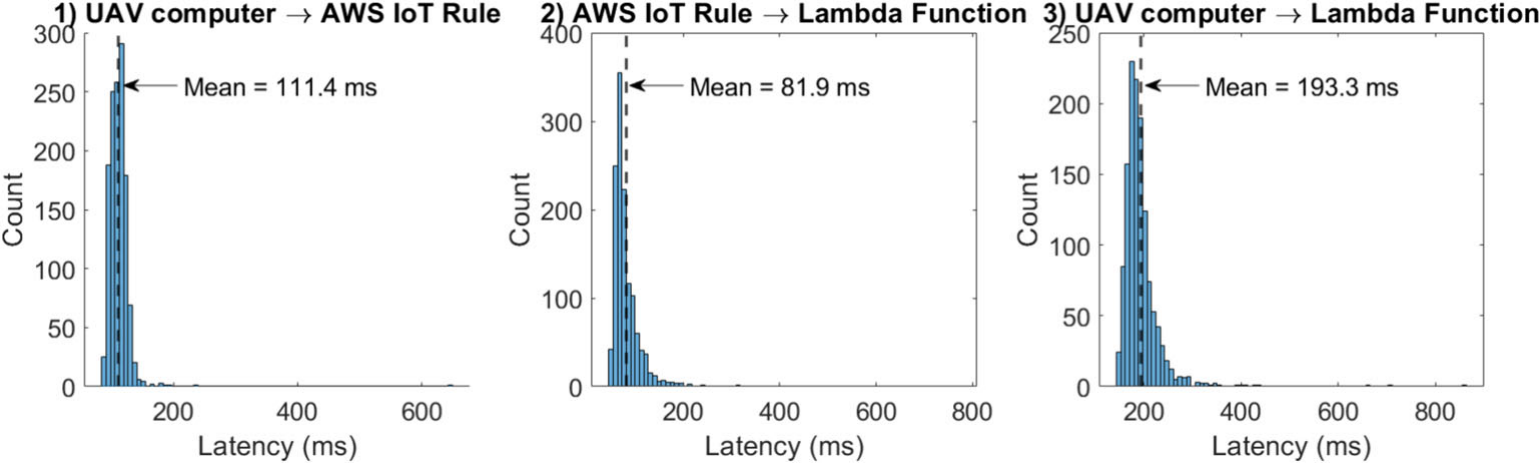
Latency measurement results for data transfer (test with 1299 packets transferred): (1) latency histogram between the data packet being sent by the drone’s onboard computer and its arrival in the AWS cloud, processed by AWS IoT Core. (2) latency histogram between processing in AWS IoT Core and processing by the lambda function for sending data to the S3 repository and calculating arrival times. (3) total latency histogram

To evaluate the performance of the MQTT-based data transmission pipeline, a latency measurement methodology based on synchronized timestamps was implemented, and the latency was computed via the AWS Lambda function service. During each acquisition cycle, the UAV’s onboard computer assigns a timestamp at the instant the data packet is published to the MQTT topic, denoted as $t_{send}$. Upon arrival at AWS IoT Core, a second timestamp $t_{IoT}$ is associated with the message, and a third timestamp $t_{L}$ is recorded when the AWS Lambda function initiates processing. Based on these timestamps, the latency between the UAV’s onboard computer and AWS IoT Core is defined as

6 $$\begin{aligned} L_{UAV \rightarrow IoT} = t_{IoT} - t_{send} \end{aligned}$$

The latency between AWS IoT Core and the Lambda function is defined as

7 $$\begin{aligned} L_{IoT \rightarrow Lambda} = t_{L} - t_{IoT} \end{aligned}$$

And the total end-to-end latency from the UAV’s onboard computer to the Lambda function is

8 $$\begin{aligned} L_{Total} = t_{L} - t_{send} \end{aligned}$$

Such that

9 $$\begin{aligned} L_{Total} = L_{UAV \rightarrow IoT} + L_{IoT \rightarrow Lambda} \end{aligned}$$

This approach is consistent with the automated data acquisition and transmission process described in the “The automated collection process” section, in which each data packet is timestamped onboard and transmitted to the cloud via MQTT. To ensure temporal consistency, the UAV’s onboard computer clock was synchronized with a network time protocol (NTP) server, while the cloud-side timestamps relied on AWS-managed time infrastructure.

A data transfer experiment to verify latency was performed, with a total of 1299 data packets transmitted, and the resulting latency distributions were statistically evaluated. The results, presented in Fig. 10, indicate a mean latency of 111.4 ms for the communication between the UAV’s onboard computer and AWS IoT Core, and 81.9 ms for the processing between AWS IoT Core and the Lambda function, resulting in a mean total end-to-end latency of 193.3 ms. The observed latency distributions are right-skewed, with most packets concentrated at low latency and a limited number of higher-latency events. These variations are attributed primarily to fluctuations in the 4G communication link and transient delays in cloud processing.

Overall, the results demonstrate that the proposed architecture enables near-real-time data transmission and processing, with latency well within the range required for continuous environmental monitoring applications.

## Discussion

The results presented in this study must be interpreted in light of important measurement limitations. Although calibration procedures and corrections were applied, no co-located validation with a reference-grade instrument was performed, and therefore, the residual uncertainty of the corrected measurements could not be quantified. In addition, the methane sensor used in this work is not suitable for detecting background atmospheric concentrations and can only identify elevated concentrations. These constraints imply that the reported measurements should not be considered as fully validated atmospheric observations. Instead, the primary contribution of this study lies in the development and demonstration of an integrated workflow for automated data acquisition, processing, and transmission. Accordingly, the results should be interpreted as a system-level proof of concept, and further work is required to achieve validated atmospheric quantification.

Within these limitations, the experiments were conducted to demonstrate the feasibility of the proposed system for automated GHG data acquisition and transmission. The results show that the system is capable of performing synchronized data collection and cloud transmission under the tested conditions, supporting the integration between onboard sensing, edge processing, and cloud-based data infrastructure.

The data resulting from the experiments, stored in the Amazon Digital repository, is raw data transmitted by the computer embedded in the drone, plus an additional column containing the $CO_{2,dry}$ calibration results for the water vapor effect described in the “Data processing” section. Moreover, the results presented in the “Current test results” section were also corrected using this methodology. The data are openly available; the links are in the Data availability statement section.

The current study addressed the automation of GHG data collection, disregarding data precision, especially when compared with data obtained with sensors commonly used in GHG monitoring, such as the well-known Los Gatos Research sensors (now ABB). The intention was to design and consolidate a process that could apply across different biomes and situations, each of which demands different levels of precision. Thus, the design goal was to establish a minimum precision for each situation with a minimum payload. For further work, we plan to explore the technological barriers to achieving a similar process with a drone capable of a larger payload and greater autonomy. Such a requirement is important to provide a target process adaptable to different biomes, such as urban regions, plantations, or offshore systems, adjusting only a few parameters or hardware support (basically sensors). The payload comprises the sensors (5 kg for the new version of ABB/Los Gatos, but ABB just launched a new version with about 3 kg), the batteries (about 1.5 kg), and an edge computer.

Satellite communication was not a key issue in the current work since different user situations could use 4G/5G wireless IP connections, either directly or using amplifiers. The requirements for communication can evolve with VLEOS (Very Low Earth Orbit Satellites), expected to be launched by 2026^6^.

Our initial motivation was to design a general process for automating GHG collection with UAVs that sends data to a cloud data space, which an intelligent orchestrator monitors. This general application would require transporting UAVs by truck or boat to the target region, avoiding the very expensive UAVs that require a large payload-to-power ratio (either batteries or fuel). Once placed at the takeoff point, the mission could be planned and launched.

Therefore, to be practical, it would require several missions at different times and/or in different locations. To address that, an important feature is minimizing the overall process cost, including all devices. Minimum cost also opens the possibility of making a data collection with more than one UAV. The cloud repository orchestrator can manage the creation of all captured datasets.

Experiments were conducted with continuous transmission to a data space during flight using an LTE/4G internet connection. Intermittence can be a drawback if the UAV flies far from the collector. However, in urban areas, there are already amplifying devices that can improve signal quality up to 5G and operate over wider areas. Alternatively, the acquired data can be stored on an onboard edge computer that can also manage the packages. This feature was important for comparing the collected data with that transmitted directly to the cloud.

The evaluation of the data transmission performance indicates that near real-time communication is achievable under the tested conditions. However, the results also reveal important limitations. While most experiments demonstrated successful data delivery with low latency, one test exhibited substantial packet loss due to a temporary communication failure. In addition, mechanisms to detect connection interruptions and retransmit missing data are not yet implemented in the current system. Therefore, the transmission performance demonstrated in this study should be interpreted as indicative of feasibility rather than reliability. Further work is required to develop and validate robust communication and data-recovery strategies to ensure consistent operation across varying network conditions.

Previous UAV-based GHG studies have achieved high measurement accuracy and emission quantification using advanced sensors (Allen et al., 2019; Kunz et al., 2020; Shaw et al., 2021). However, the primary contribution of this study differs in scope and objective. This work focuses on automating and scaling the entire data acquisition and management pipeline rather than maximizing measurement accuracy. Specifically, we propose and demonstrate an end-to-end system that integrates in-flight data acquisition, onboard preprocessing, near real-time transmission over 4G, and automatic ingestion into a cloud-based data space (Digital Amazon), thereby eliminating manual data-handling steps. The key advantages of this study compared to previous work are as follows:

- End-to-end automation of GHG data collection and transmission in near real time
- Integration with a cloud-based data infrastructure, including metadata, versioning, and standardized datasets
- A low-cost and scalable approach, enabling potential deployment of multiple UAVs for large-area monitoring
- A system-level architecture designed for operational and continuous environmental monitoring

Therefore, while prior studies emphasize measurement accuracy and emission quantification, this work advances the field by addressing the operationalization, scalability, and data infrastructure required for continuous UAV-based GHG monitoring systems.

## Conclusion

In conclusion, a flexible process to automate GHG and other gas emissions is feasible, transferring data continuously to the cloud. Medium-sized UAVs, even a fleet, could collect greenhouse gas data and transmit it to a cloud system called Digital Amazon, which would integrate it into a single dataset. The proposition has been discussed with several UAV manufacturers to assess its feasibility for broader implementation. The main drawback—the payload—is no longer a challenge thanks to the evolution of sensors that combine high precision with an optimized power source and good calibration. Thus, the results are scalable, feasible, and have potential for application in other ecosystems.

Therefore, the process explored in this work offers a feasible alternative for environmental monitoring. GHG mapping was tested across a survey area or multiple altitude levels using an agile, flexible approach, but was limited to the core automation of the monitoring process.

However, the automation of the GHG data collection and transmission process enabled environmental online monitoring systems and the identification/localization of emission sources, such as fires, through the application of source identification and location algorithms, processed on board the UAV or via a computer connected to the cloud.

Further work should focus on enhancing the automation process by introducing intelligent control of the UAV mission, optimizing the UAV flight path to detect gas-emitting sources, and applying new source-detection and localization algorithms. To further work, we intend to test the present proposal to automate GHG data collection applied to the Atlantic forest and other strategic environments, such as large plantations and urban areas.

## Data Availability

The original data presented in the study (the 9 flight tests, being 8 missions of measuring $CO_2$ concentration along altitude and one mission to measure CO2 concentration mapping over survey area) are openly available in Digital Amazon at: https://www.rcggi.org/en/data_lake/datasets/details?code=7c797613-f179-4fb4-b62f-77fda529dff2 https://www.rcggi.org/en/data_lake/datasets/details?code=75f775b3-45f1-4fe5-aa83-8b57c953a6f5 https://www.rcggi.org/en/data_lake/datasets/details?code=047a1e4f-68f0-4812-999c-8ef4a19e425f https://www.rcggi.org/en/data_lake/datasets/details?code=87e8e0f9-500e-4d06-90a7-5a246526a190 https://www.rcggi.org/en/data_lake/datasets/details?code=2d9d52d2-7463-4257-8c98-d87b5bf4d17e https://www.rcggi.org/en/data_lake/datasets/details?code=da565d41-b397-4bdd-9376-3cef875f24b0 https://www.rcggi.org/en/data_lake/datasets/details?code=4e4aa802-77aa-405d-bfde-38c46cdccec4 https://www.rcggi.org/en/data_lake/datasets/details?code=5bdbee63-0395-49ea-ab19-ecd4f0c99d87 https://www.rcggi.org/en/data_lake/datasets/details?code=d849df21-3448-4bf4-a411-2de3c4a73a12

## Footnotes

1 PX4 Autopilot, https://docs.px4.io/main/en/

2 https://github.com/dronekit/dronekit-python

3 Python ULID, https://pypi.org/project/python-ulid/

4 Flight Review, https://review.px4.io/

5 Matlab, https://www.mathworks.com/products/matlab.html

6 https://eda.europa.eu/news-and-events/news/2024/03/06/european-satellite-constellation-for-very-low-earth-orbit-to-launch-in-two-years
